# The effects of royal jelly supplementation on anthropometric indices: a GRADE-assessed systematic review and dose-response meta-analysis of randomized controlled trials

**DOI:** 10.3389/fnut.2023.1196258

**Published:** 2023-08-03

**Authors:** Mahdi Vajdi, Vali Musazadeh, Mahsa Khajeh, Ehsan Safaei, Melika Darzi, Nooshin Noshadi, Hadi Bazyar, Gholamreza Askari

**Affiliations:** ^1^Student Research Committee, Isfahan University of Medical Sciences, Isfahan, Iran; ^2^Student Research Committee, Tabriz University of Medical Sciences, Tabriz, Iran; ^3^School of Nutrition and Food Sciences, Tabriz University of Medical Sciences, Tabriz, Iran; ^4^Department of Clinical Nutrition, School of Nutrition and Food Sciences, Nutrition Research Center, Tabriz University of Medical Sciences, Tabriz, Iran; ^5^Department of Nutrition, Science and Research Branch, Islamic Azad University, Tehran, Iran; ^6^Student Research Committee, Sirjan School of Medical Sciences, Sirjan, Iran; ^7^Department of Public Health, Sirjan School of Medical Sciences, Sirjan, Iran; ^8^Department of Community Nutrition, School of Nutrition and Food Science, Nutrition and Food Security Research Center, Isfahan University of Medical Science, Isfahan, Iran

**Keywords:** royal jelly, supplementation, weight, BMI, obesity, meta-analysis

## Abstract

Inconsistent data are available about the effect of royal jelly supplementation on anthropometric indices in humans. This systematic review and meta-analysis was done to summarize data from available randomized controlled trials (RCTs) on the effect of royal jelly supplementation on anthropometric indices such as body weight (BW), body mass index (BMI), and fat mass (FM) in adults. We systematically searched Embase, PubMed, Web of Science, and Scopus databases up to March 2023. All RCTs assessing the effect of royal jelly on anthropometric indices were included. Data were pooled using the random-effects method and were expressed as weighted mean difference (WMD) and 95% confidence intervals (CIs). Sensitivity and subgroup analyses were also performed. Out of 1,492 records, 10 studies that enrolled 512 participants were included. There was no significant effect on BW (WMD: −0.29 kg, 95% CI: −1.24, 0.65, *p* = 0.543), BMI (WMD: 0.11 kg/m^2^, 95% CI: −0.29, 0.52, *p* = 0.583), and FM (WMD: 0.02%, 95% CI: −0.41, 0.46, *p* = 0.84). However, we observed a reduction in BW and BMI following royal jelly intake in subgroup of royal jelly dosage <3,000 mg/day. Although the royal jelly supplementation significantly reduced BW and BMI at the dosages <3,000 mg/day, until additional trials have been conducted to assess the effects on obesity measures, it is best to prescribe royal jelly with caution.

## Introduction

Obesity is a complex disease condition that indicate abnormal accumulation of body fat and lead to adverse health effects (1, 2). Global prevalence of obesity has increased substantially over the past 40 years, from less than 1% in 1975, to 6–8% in 2016 (3). It affects the quality of life, increases the risk of several diseases such as type 2 diabetes mellitus (T2DM), cardiovascular diseases (CVDs), and some types of cancer (4, 5). Consequently, weight management approaches are of great importance to improve the physical and mental health of obese patients. Although several researchers have examined the preventive strategies for obesity, development of novel approaches to prevent the progression of obesity and overweight is of great clinical importance. In recent years, the use of natural products has been considered along with traditional methods including lifestyle modification through physical activity and proper diet in the weight management (6–8). Furthermore, natural products are easily available, economical, and are relatively safe the royal jelly is one of them (9–11).

The royal jelly is a viscous and milky substance produced by the young working bees and contains vitamins, minerals, water (60–70%), proteins (12–15%), carbohydrates (10–12%), and lipids (3–7%) (9–12). 10-hydroxy-2decenoic acid (10-HDA), a well-known bioactive substance of royal jelly, plays important roles in several biological processes, including oxidative stress and inflammation (13). The royal jelly is now widely consumed as a health food supplement and in cosmetic products and is as a functional food of interest for the improvement of human being (14). Several studies have indicated that proteins of royal jelly are a major contributor for several physiological functions (15, 16). The proteins in royal jelly have antioxidant activities and it is being used as an anti-aging agent and as part of the treatment of hypertension, asthma, atherosclerosis, diabetes and depression which are caused by oxidative stress (17). Royal jelly supplementation can be considered as an adjuvant therapy for the treatment of a wide range of diseases due to its anti-inflammatory, antitumor, anti-allergic, antioxidant, immunomodulatory, hypotensive, insulin-like properties (18–23). It has been supposed that anti-oxidative peptides derived from royal jelly proteins hydrolyze with protease N to produce the robust anti-oxidative effect (24). Several studies proposed that royal jelly may be useful in management of body weight (BW) because it has favorable effect on oxidative stress, inflammation, dyslipidemia, and insulin resistance (23, 24). Moreover, royal jelly exerts helpful effects on pancreatic lipase activity and energy metabolism (25, 26).

Several studies reported conflicting effects of royal jelly supplementation on body composition; however the reason or underlying mechanism for improving body composition was not fully explored. Some studies reported that royal jelly supplementation had a favorable effect on body composition (27, 28). In contrast, several studies indicated that royal jelly could not improve anthropometric indices (29–31). However, despite several studies on the effect of royal jelly intake on anthropometric indices, we are aware of no prior study summarizing results in this regard. The present study was therefore done to perform a comprehensive systematic review and meta-analysis of published randomized controlled trials (RCTs) to evaluate the effect of royal jelly supplementation on BW, body mass index (BMI), and fat mass (FM) in adults.

## Materials and methods

This meta-analysis was conducted according to Preferred Reporting Items for Systematic Reviews and Meta-Analyses (PRISMA) guidelines (32) (Supplementary Table S1) and recorded in the International Prospective Register of Systematic Reviews (PROSPERO) with code CRD42023409546. The protocol of the present study has been approved by the ethics committee of Isfahan University of Medical Sciences (identifier: IR.MUI.RESEARCH. REC.1402.014 and grant number: 140209).

### Search strategy

We systematically searched electronic databases including Embase, PubMed, Web of Science, and Scopus from inception through March 2023 with the following terms: royal jelly OR royal jelly AND obesity OR weight OR abdominal obesity OR BMI OR adipose tissue OR Body Mass Index OR visceral obesity OR Overweight OR fat mass OR WC OR waist circumference OR WHR OR waist to hip ratio OR adiposity. Searches of the databases did not include language or date restrictions. Moreover, in our manual search of the references, we were able to identify additional studies that were not identified by our database search.

### Study selection

Our criteria for including studies were as follows: (1) a parallel or crossover design RCTs, (2) study conducted on adult participants, (3) investigated the effects of royal jelly supplementation on anthropometric indices, and (4) study designs that include enough data to assess outcomes at both baseline and post-intervention. We excluded studies based on these criteria: (1) studies without a placebo or control group, (2) trials in which children or pregnant women were enrolled, and (3) conference abstracts, *in vivo* and *in vitro* studies, reviews, letters, or case reports.

### Data extraction

Independently, two researchers (MV and MK) extracted data from eligible studies. In order to reach a consensus, a chief investigator (GA) evaluated any inconsistencies. Data included were the following: first author’s names, publication year, location of study, duration of the study, royal jelly dosage, type of royal jelly and placebo, mean age, study design, participants per group, gender, participants’ health status, and main outcome.

### Quality assessment and certainty of evidence

An independent assessment of the methodological quality and bias risk of the trials included in the meta-analysis was carried out by two authors (MV and ES), according to the Cochrane criteria (33). Seven criteria were used to assess the included studies: Random sequence generation adequacy, personnel’s and participants’ blinding, outcome assessment, allocation concealment, incomplete outcome data, selective reporting, and other possible bias causes (33). Moreover, we used the GRADE method to grade the overall certainty of evidence across the studies (34). Depending on the evaluation criteria, evidence quality could be classified into four categories: very low, low, moderate, and high (35).

### Statistical analysis

In order to conduct the meta-analysis, Stata (StataCorp, College Station, TX, United States) version 14 was used. We calculated the overall estimates based on the means and SDs reported for the intervention and control groups (36). Each variable’s effect size was listed as a weighted mean difference (WMD) and 95% confidence intervals (CIs) (37). We calculated the pooled effect size using a random-effects model in the presence of heterogeneity. In order to assess heterogeneity in the included studies, a Cochran’s Q test and *I*^2^ statistic were used (*I*^2^ > 50% is considered considerable heterogeneity) (33). To detect the sources of heterogeneity, subgroup analyses were carried out based on the duration of intervention, the dosage of royal jelly supplementation, gender, participants’ health condition, sample size, and quality of studies. Using a sensitivity analysis, we evaluated the impact of each study on the overall effect size. The funnel plot inspection and Egger’s test were used to determine publication bias. We also applied fractional polynomial modeling to determine the non-linear potential effects of royal jelly dosage and treatment duration on anthropometric indices (38).

## Results

### Study selection

**Figure 1:**
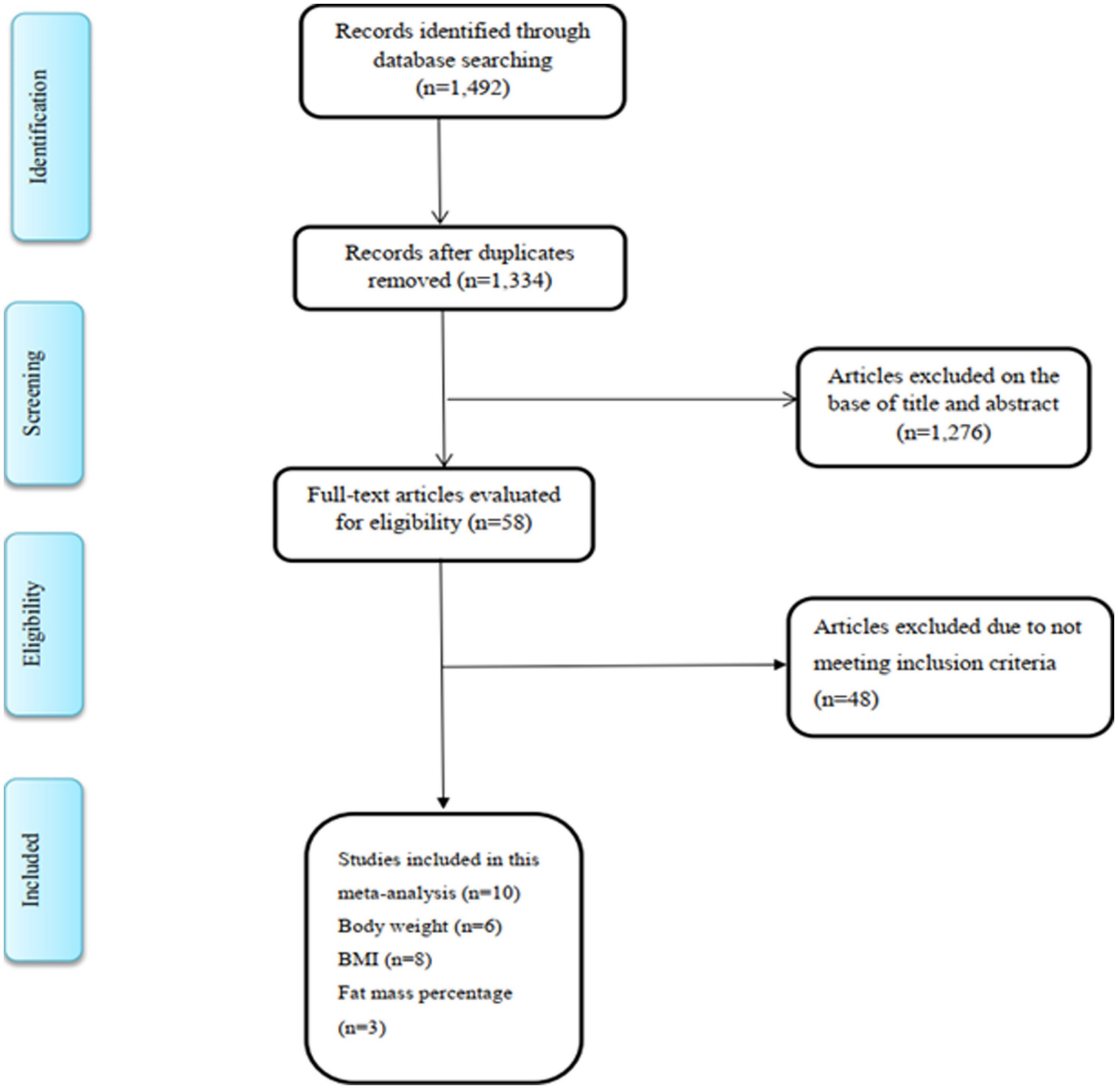
Flow diagram of study screening and selection process.

In the initial search, after removing duplicate studies, 1,492 studies were identified. On the basis of title and abstract, we omitted 1,276 irrelevant studies and 58 studies were chosen for further assessment and detailed examination. In full-text screening, 48 studies were excluded based on the abovementioned inclusion criteria. Finally, 10 eligible studies satisfied the inclusion criteria and were included in the final analysis (10, 17, 28–31, 39–42) (Figure 1). Out of 10 studies, six studies examined the effect of royal jelly on BW, eight reported data on BMI, and three studies had reported data on FM.

### Study characteristics

The general characteristics of eligible studies are summarized in Table 1. The sample size of the included studies totaled 512, ranging from 15 to 88 participants in individual studies. Included studies were published from 2007 to 2022, and individuals’ age range in these studies was between 21 and 62 years. Studies were carried out in Iran (28, 31, 40, 41), Brazil (30), Turkey (17), Taiwan (29), Japan (10, 39), and Slovenia (42). The range of dosage of royal jelly supplements varied from 500 to 6,000 mg/day and the duration of intervention was between 4 and 18 weeks in different trials. All records examined the effect of royal jelly in both genders except for two studies that were restricted to men (17) and women (28). Included studies had recruited people with diabetes (28, 31, 40, 41), overweight adults (42), and apparently healthy subjects (10, 17, 29, 30, 39). In addition, studies were performed in subjects with different baseline BMI; four studies were carried out in subjects with normal BMI (BMI < 24.9 kg/m^2^) (10, 17, 30, 39), four studies (28, 31, 40–42) in overweight subjects (25 kg/m^2^ < BMI < 29.9 kg/m^2^) and one other did not report BMI (29). Among the included studies, only four studies by Chiu et al. (29) (carbohydrate 70 mg, protein 50 mg, fat 10 mg, and sodium 0.19 mg), Petelin et al. (42) (organic lyophilised royal jelly, standardized to a minimum of 4% 10-HDA), Pourmoradian et al. (28) (lyophilized royal jelly), and Fujisue et al. (39) (protein 11.4%, moisture 66.9%, sugar 9.1%, ether extract 6.2%, and ash 0.94%) reported the components of royal jelly. Moreover, the type of placebo was also varied between trials. Three studies administered glycerin (31, 40, 41), whereas the rest of studies administered different type of placebo such as corn starch (17, 29), liquid without royal jelly (10), soft gel (28), rice starch (42) and maltose syrup, cellulose, and sucrose fatty acid ester (39). In terms of changes in energy intake, a significant reduction was observed in one study (28), while four studies did not find such an effect (31, 40–42) and five other did not report energy intake (10, 17, 29, 30, 39). Moreover, the results of all studies were not adjusted for confounding factors which can affect anthropometric indices such as energy intake, physical activity, and usual dietary intakes of vegetables, and fruits.

**Table 1:** Characteristics of eligible studies on the effects of royal jelly supplementation on anthropometric indices in adults.

References	Year/country	Study design	Subject	Sample size (intervention/control)	Mean age (intervention/control)	Baseline BMI (intervention/control)	Duration (week)	Intervention		Main outcome
								Treatment group (g/d)	Control group	
Guo et al. (30)	2007/Brazil	RCT	Healthy	15 (7/8)	39/36.9	21.85 (21.9/21.8)	4	6,000	Nothing	No significant change in BW, BMI and FM
Cicero et al. (43)	2011/Turkey	RCT	Healthy	40 (30/10)	21.5	21.94 (22.53/21.36)	4	500/1,000/2,000	Placebo (corn starch)	No significant change in BW, BMI and FM
Pourmoradian et al. (28)	2012/Iran	RDBPC	T2DM	45 (23/22)	51.71/51.44	28.48 (28.98/28.7)	8	1,000 (lyophilized royal jelly)	Placebo (soft gel)	Significant decrease in energy intake, and BW
Morita et al. (10)	2016/Iran	RDBPC	T2DM	46 (23/23)	51.78/53.13	27.9 (27.79/28.01)	8	3,000	Placebo (glycerin)	No significant change in energy intake, BW and BMI
Shidfar et al. (31)	2016/Taiwan	Single-blind RCT	Mild hypercholesterolemic	40(20/20)	–	–	12	3,150 (protein 50 mg, fat 10 mg, carbohydrate 70 mg and sodium 0.19 mg)	Placebo (corn starch)	No significant change in BW, FM, and WC
Morita et al. (10)	2012/Japan	RDBPC	Healthy	56 (30/26)	62.5	22.75 (22.7/22.8)	24	3,000	Placebo (liquid without royal jelly)	No significant change in BMI and WC
Amini et al. (44)	2015/Iran	RDBPC	T2DM	46 (23/23)	51.8/53.13	27.9 (27.79/28.01)	8	3,000	Placebo (glycerin)	No significant change in energy intake, and BMI
Mousavi et al. (41)	2017/Iran	RDBPC	T2DM	46 (23/23)	–	–	8	3,000	Placebo (glycerin)	No significant change in energy intake, and BW
Chiu et al. (29)	2019/slonenia	RDBPC	Overweight	60 (30/30)	41.1	26.95 (26.2/27.7)	8	666 (organic lyophilized royal jelly, standardized to a minimum of 4% 10-HAD)	Placebo (rice starch)	No significant change in energy intake, BMI, FM
Fujisue et al. (39)	2022/Japan	RDBPC	Healthy	88 (46/42)	36.1/35	22.35 (22.3/22.4)	4	690 (protein 11.4%, moisture 66.9%, sugar 9.1%, ether extract 6.2%, and ash 0.94%)	Placebo (maltose syrup, cellulose, and sucrose fatty acid ester)	No significant change in BMI

BW, body weight; BMI, Body Mass Index; FM, fat mass, T2DM Type 2 diabetes, RCT, randomized controlled trial, RDBPC, randomized, double-blind, placebo-controlled trial.

**Figure 2:**
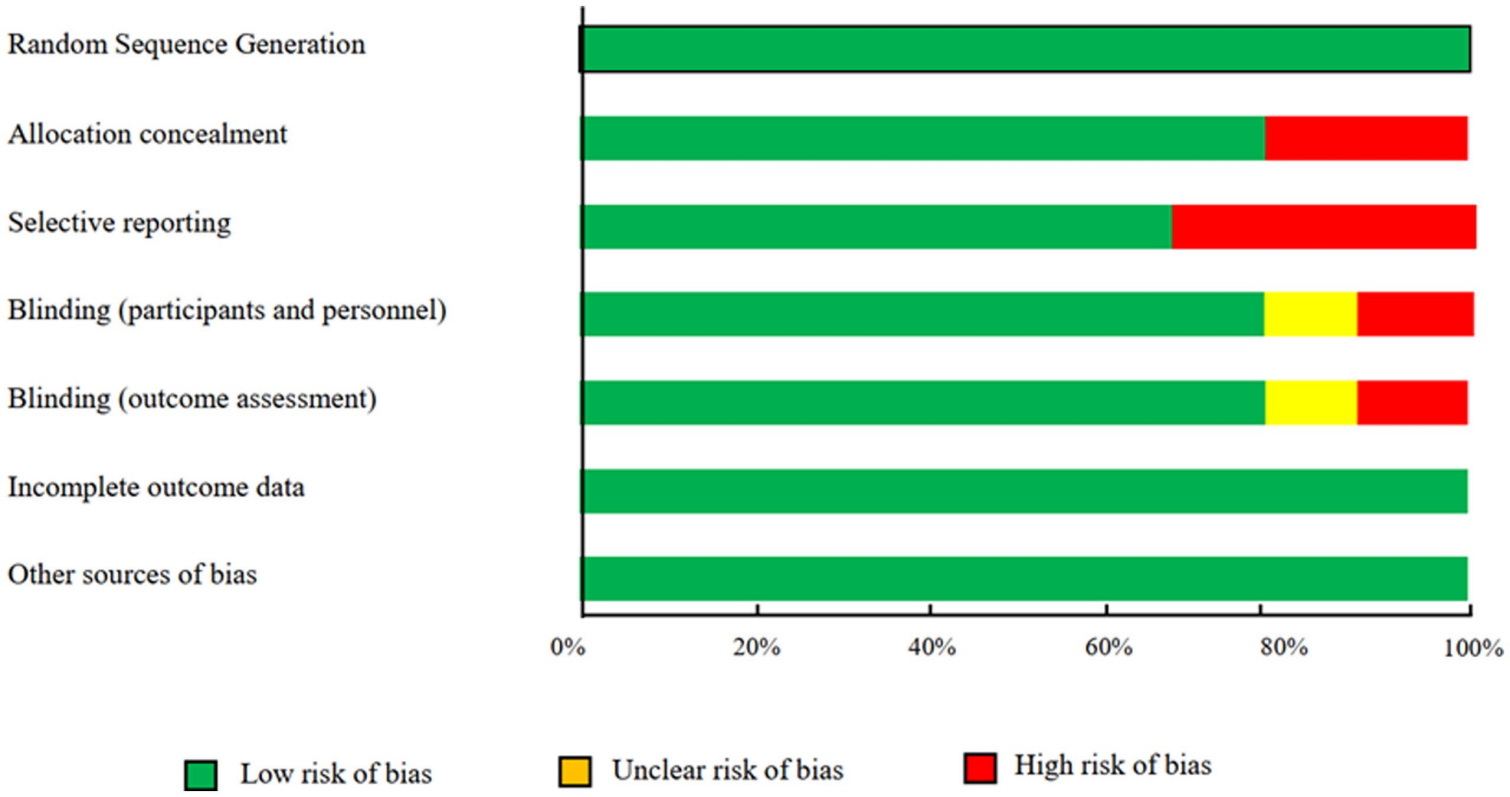
Results of risk of bias assessment for trials included in the current meta-analysis on the effects of royal jelly supplementation on anthropometric indices.

The findings of the quality assessment of included trials according to Cochrane Collaboration’s risk of bias tool are provided in Figure 2, and, overall, included trials had good and moderate quality. Most trials revealed adequate quality for key factors. All trials presented enough data about incomplete outcome data and random sequence generation. Proper blinding was observed in 80% of included studies. Two of the studies did not explain the allocation concealment procedure of the study. There was selective reporting bias in the 30% of included trials.

### Findings for the effect of royal jelly on BW

**Figure 3:**
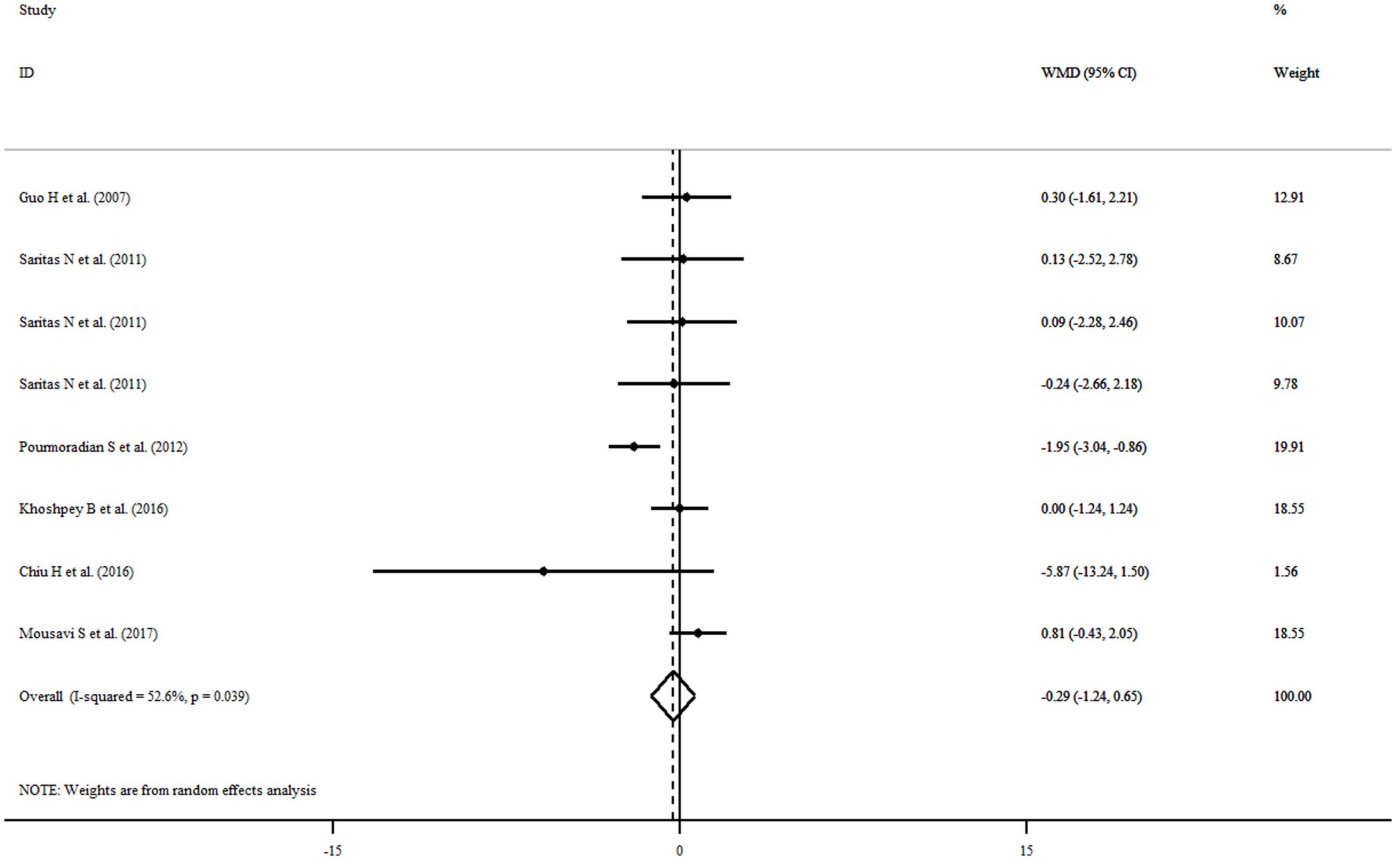
Forest plot illustrating weighted mean difference and 95% confidence intervals for the impact of royal jelly on BW.

Six eligible articles with eight treatment arms including a total of 252 participants examined the effect of royal jelly supplementation on BW. Combining findings from the random-effects model indicated that royal jelly had no significant effect on BW (WMD: −0.29 kg, 95% CI: −1.24, 0.65, *p* = 0.543) as compared to controls, with significant between-study heterogeneity (*I*^2^ = 52.6%, *p* = 0.039) (Figure 3). Subgroup analysis based on different variables indicated that duration of intervention, quality, dose, health status, gender, and sample size could explain the heterogeneity. Moreover, we observed a reduction in BW following royal jelly intake in subgroup of royal jelly dosage <3,000 mg/day (WMD: −1.23 kg, 95% CI: −2.10, −0.36, *p* = 0.005) (Supplementary Table S2). Sensitivity analysis showed that no study had a significant impact on the overall effect sizes of BW (Supplementary Figure S1). The meta-regression analysis revealed that the pooled estimate is independent of royal jelly dose (slope: 0.0002; 95% CI: −0.0003, 0.0009; *p* = 0.306) and treatment duration (slope: −0.2233; 95% CI: −0.7848, 0.3380; *p* = 0.368) (Supplementary Figures S2A,B).

### Findings for the effect of royal jelly on BMI

**Figure 4:**
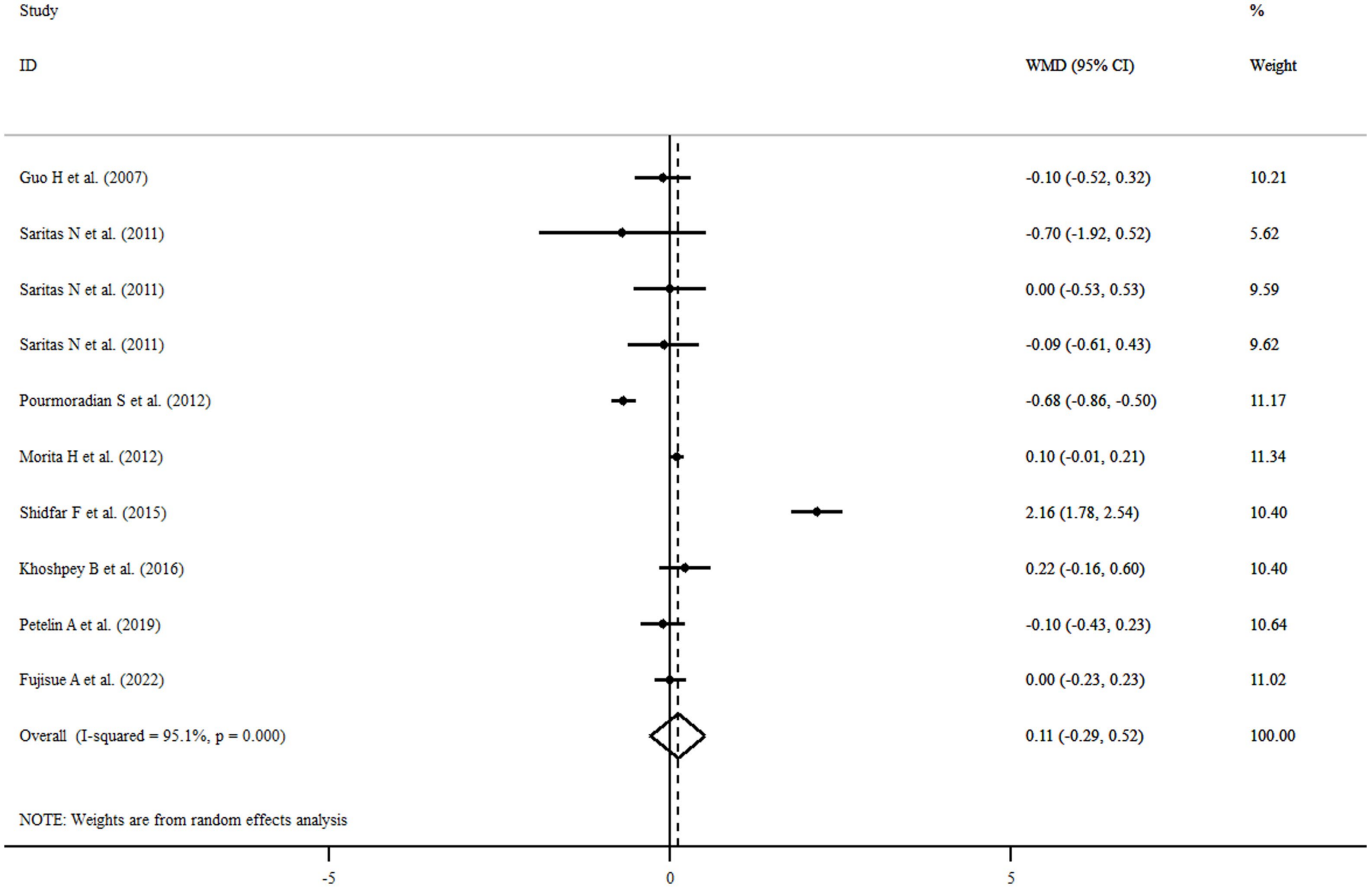
Forest plot illustrating weighted mean difference and 95% confidence intervals for the impact of royal jelly on BMI.

Among eligible articles, eight studies with 10 treatment arms including a total of 416 participants reported the association of royal jelly consumption with BMI. Royal jelly consumption did not affect BMI significantly (WMD: 0.11 kg/m^2^, 95% CI: −0.29, 0.52, *p* = 0.583) with significant heterogeneity among studies (*I*^2^ = 95.1%, *p* < 0.001) (Figure 4). Subgroup analysis based on different variables indicated that sample size, gender, age, health status, dose, duration of intervention, and quality of studies could explain the heterogeneity. Furthermore, we observed a reduction in BMI following royal jelly intake in subgroup of royal jelly dosage <3,000 mg/day (WMD: −0.33 kg/m^2^, 95% CI: −0.45, −0.21, *p* < 0.001) (Supplementary Table S3). Sensitivity analysis showed that no study had a significant impact on the overall effect sizes of BMI (Supplementary Figure S3). The meta-regression analysis revealed that the pooled estimate is independent of royal jelly dose (slope: 0.0004; 95% CI: −0.00004, 0.00091; *p* = 0.070) and treatment duration (slope: 0.0259; 95% CI: −0.1191, 0.0795; *p* = 0.1709) (Supplementary Figures S4A,B).

### Findings for the effect of royal jelly on FM

**Figure 5:**
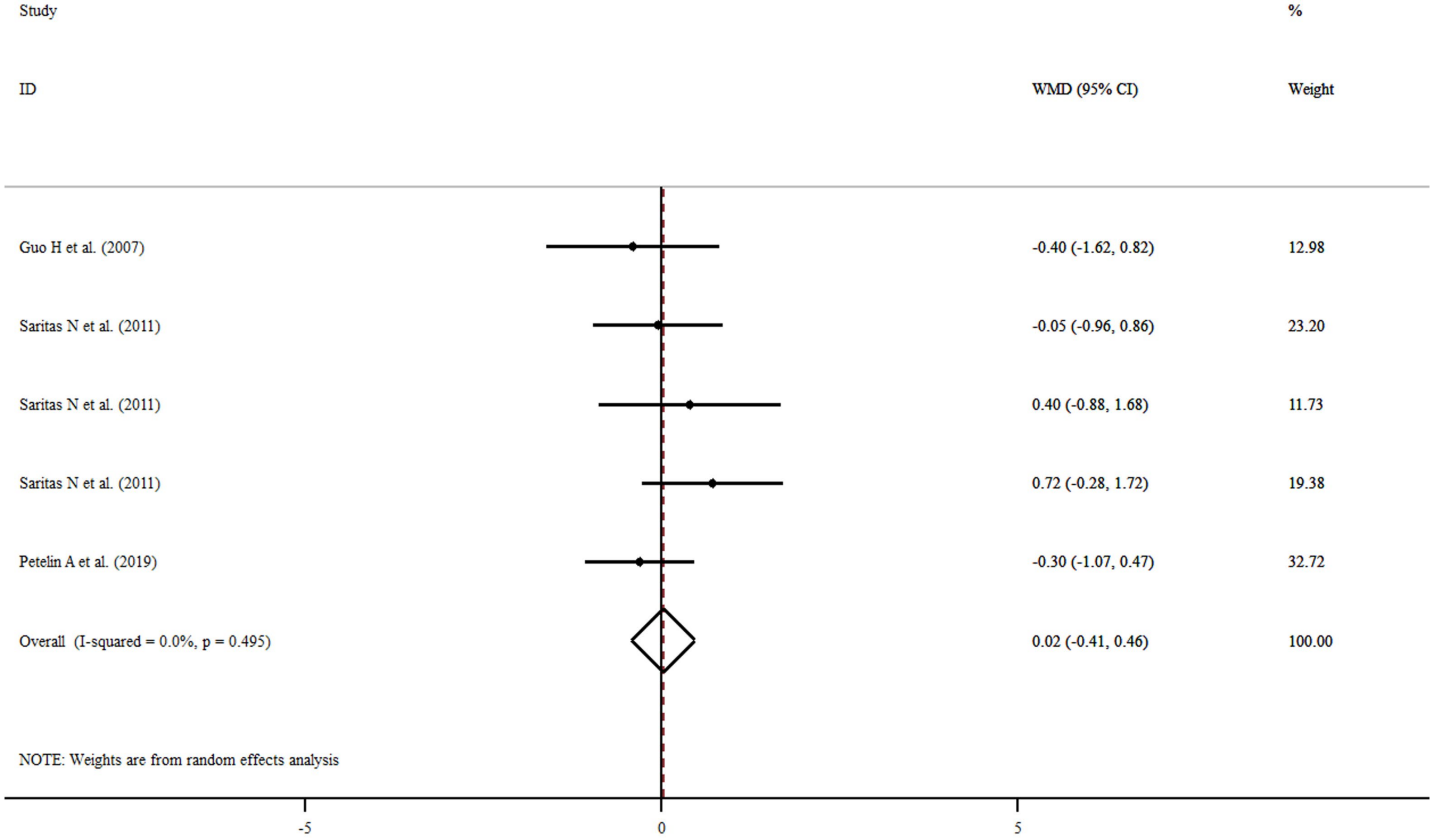
Forest plot illustrating weighted mean difference and 95% confidence intervals for the impact of royal jelly on FM.

Three studies with five treatment arms including a total of 135 participants reported FM as an outcome measure. Combined results from the random-effects model indicated that FM did not change significantly following royal jelly consumption (WMD: 0.02%, 95% CI: −0.41, 0.46, *p* = 0.84) with no observed heterogeneity across the studies (*I*^2^ = 0.0%, *p* = 0.495) (Figure 5). None of our subgroup analyses showed a significant effect of royal jelly supplementation on FM (Supplementary Table S4). Sensitivity analysis showed that no study had a significant impact on the overall effect sizes of FM (Supplementary Figure S5). The meta-regression analysis revealed that the pooled estimate is independent of royal jelly dose (slope: −0.1206; 95% CI: −0.00046, 0.00038; *p* = 0.801) and treatment duration (slope: 0.0259; 95% CI: −0.4997, 0.2583; *p* = 0.386) (Supplementary Figures S6A,B).

### Outcomes not included in the meta-analysis: WC

We found two studies on the effect of royal jelly supplementation on WC and the results showed no significant effect on WC (10, 29).

### Non-linear dose-responses between dose and duration of royal jelly supplementation and outcomes

**Figure 6:**
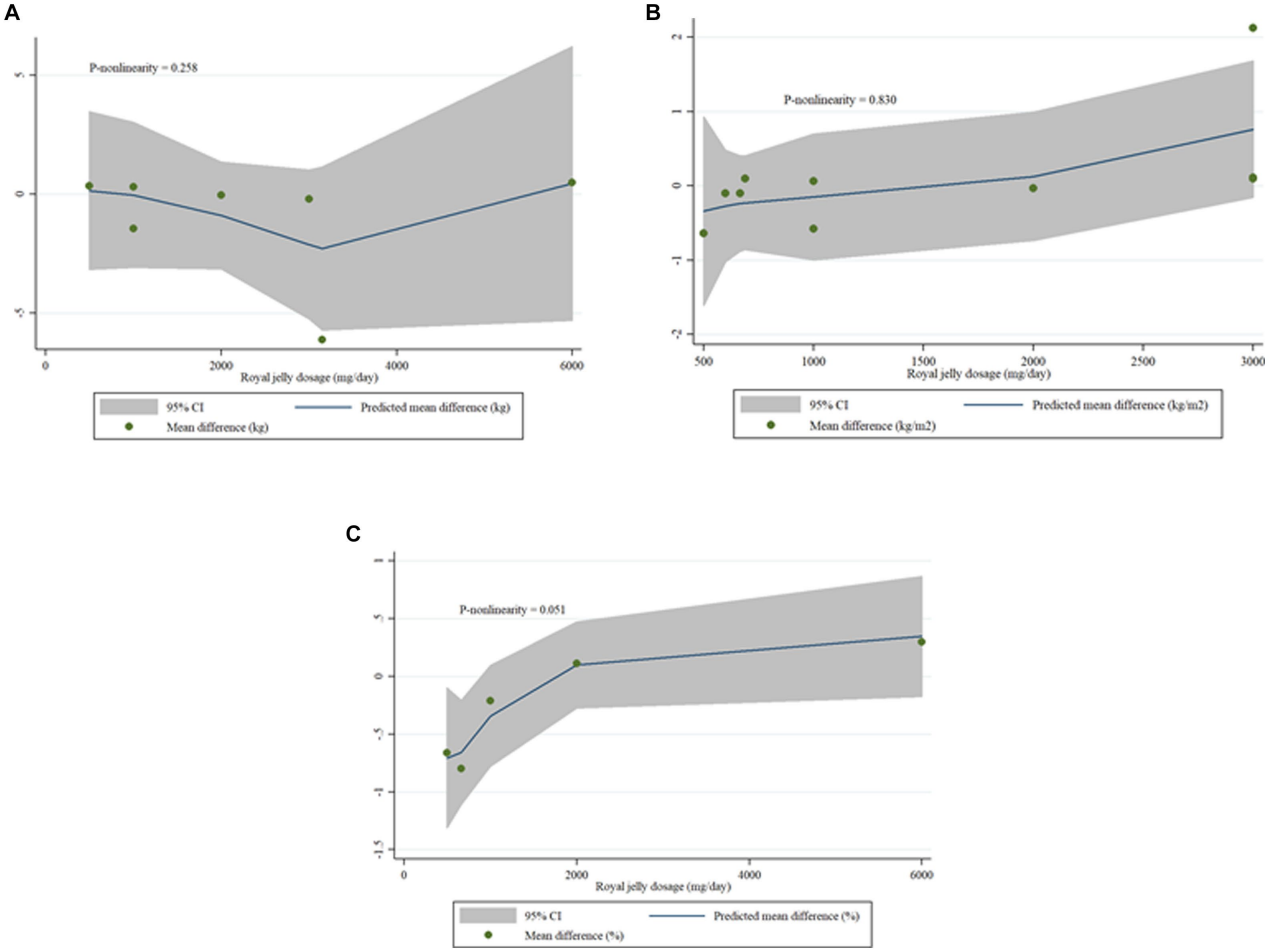
Dose-response relations between royal jelly dosage (mg/d) and mean difference in BW **(A)**, BMI **(B)**, FM **(C)**.

**Figure 7:**
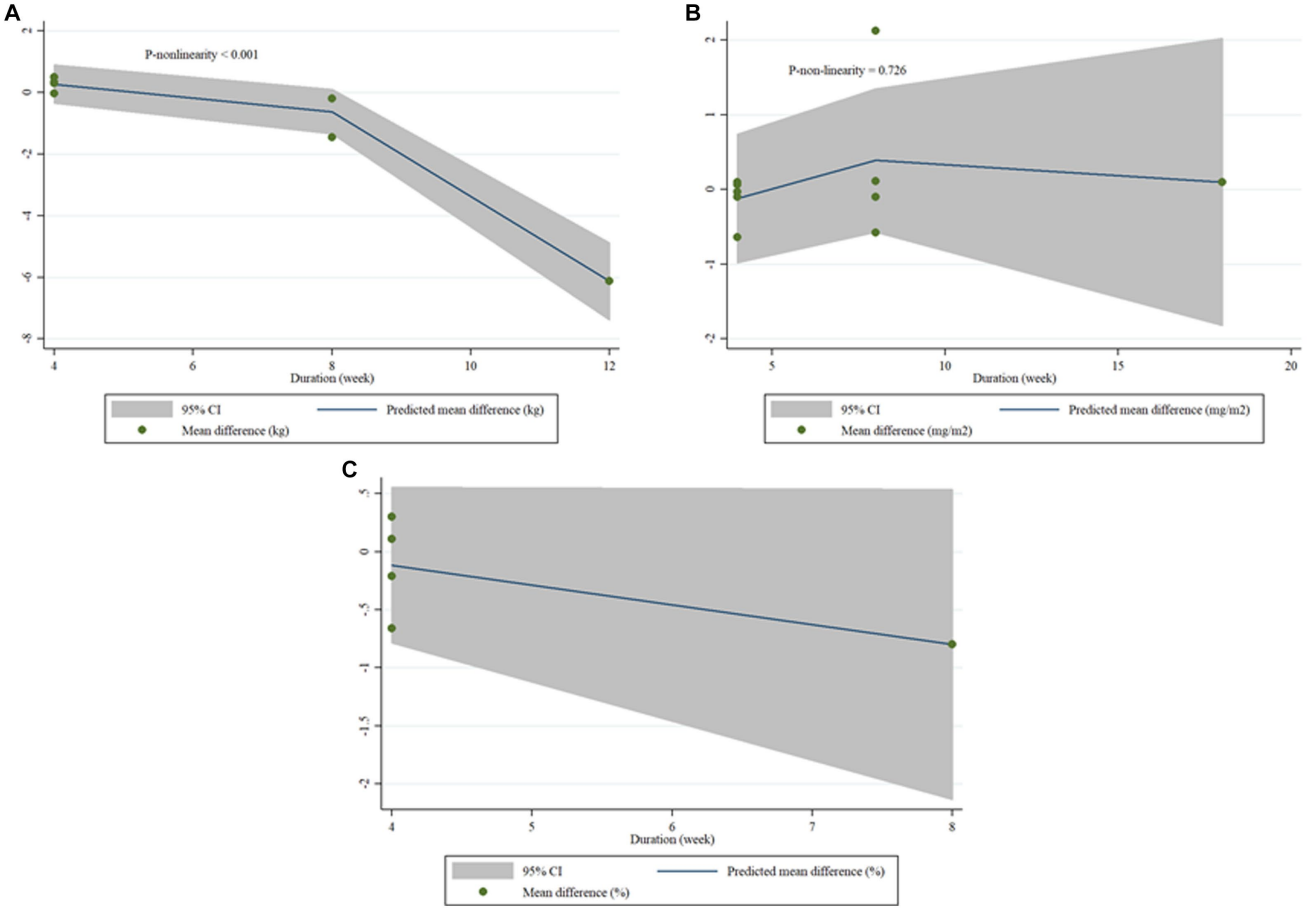
Dose-response relations between duration of royal jelly supplementation (weeks) and mean difference in BW **(A)**, BMI **(B)**, FM **(C)**.

We failed to show nonlinear dose-response effect of royal jelly on BW (P-nonlinearity = 0.214), BMI (P-nonlinearity = 0.258) as well as FM (P-nonlinearity = 0.051) (Figures 6A–C). Duration of royal jelly consumption was shown to have not a significant non-linear relationship with BMI (P-nonlinearity = 0.726) as well as FM (P-nonlinearity = 0.241). However, BW (P-nonlinearity < 0.001) changes in non-linear fashion (Figures 7A–C).

### Publication bias

Visual inspection of the funnel plots for BW, BMI, and FM did not indicate any evidence of asymmetry (Supplementary Figures S7A–C). Also, there was no publication bias among studies according to Begg’s (BW: *p* = 0.319; BMI: *p* = 0.788; and FM: *p* = 0.462) and Egger’s test (BW: *p* = 0.937; BMI: *p* = 0.714; and FM: *p* = 0.568) and visual inspection of the funnel plot.

### GRADE assessment

The overall certainty of the evidence for the effects of royal jelly supplementation on body composition is untaken in Table 2. The quality of the evidence for BW, and BMI was graded as “low” after being downgraded for inconsistency and imprecision. Meanwhile, the quality of the evidence for FM was graded as “moderate” after being downgraded for imprecision.

**Table 2:** Evaluation of the overall body of evidence by GRADE system.

Outcome	No of studies	Design	Risk of bias	Inconsistency	Indirectness	Imprecision	Publication bias	Quality of evidence
Body weight	6	RCTs	No serious^a^	Serious^b^	No serious^c^	Serious^d^	No serious^e^	Low
BMI	8	RCTs	No serious	Serious	No serious	Serious	No serious	Low
Fat mass	3	RCTs	No serious	No serious	No serious	Serious	No serious	Moderate

The quality of evidence is divided into 4 levels using GRADE system (high, moderate, low, very low).

a For risk of bias, the majority of included studies were considered to be at low risk of bias.

b Downgraded if there was a substantial unexplained heterogeneity (*I*^2^ > 50%, *p* < 0.10) that was unexplained by meta-regression or subgroup analyses.

c Downgraded if there were factors present relating to the participants, interventions, or outcomes that limited the generalizability of the results.

d Optimal information size was not met, or the 95%CI include the null value lower and upper bounds of the 95%CI were <0.95 and >1.05, respectively.

e Downgraded if there was an evidence of publication bias using funnel plot.

## Discussion

In the current systematic review and meta-analysis of available 10 RCTs, we summarized the available evidence on the effects of royal jelly supplementation as a dietary approach on anthropometric indices. The results obtained from the pooled analysis suggested that royal jelly supplementation, in comparison with control, exerted no significant influence on BW, BMI, and FM. Further, based on subgroup analysis, our results revealed a significant reduction in BW and BMI following royal jelly intake in subgroup of royal jelly dosage <3,000 mg/day. Also, meta-regression analysis indicated that duration and dosage of royal jelly supplementation were not significantly associated with anthropometric indices.

The prevalence of obesity is rising drastically over the world. Despite numerous studies and efforts, the prevalence of obesity continues to rise in both industrialized and developing nations, showing that the present weight management measures are inadequate. Hence, finding an appropriate approach remains a major concern (44). Dietary components that have medicinal or therapeutic advantages are considered as variables in the management of chronic diseases, such as obesity (7, 43). In this regard, royal jelly, due to its unique composition, is considered as an interesting functional food (19, 28). Significant discussion has surrounded the influence of royal jelly supplementation on anthropometric indices. According to the results obtained from pooled analysis, royal jelly supplementation could not significantly enhance anthropometric indices. However, the results of subgroup analysis suggested that royal jelly could lead to a significant decrease in BW and BMI in subgroup of royal jelly dosage <3,000 mg/day. The discrepancy observed in the outcomes might be attributed to a number of reasons. This might be explained by higher sample size or high number of the included studies in this subgroup, which provided increased statistical power to detect significant effects. Further, the null outcome obtained from our analysis might be due to the fact that the people recruited in the included RCTs were normal weight or overweight. There is a possibility that royal jelly can exert its optimum influence in obese patients. Besides, it is noteworthy to mention that the variability in the quality of royal jelly products used in different studies is an important consideration that may impact the nutritional content and bioactive profile of the product, potentially affecting the outcomes of intervention studies. We acknowledge that there is a wide range of variability in the composition of royal jelly products due to factors such as geographic location, season, bee species, and processing methods (45). Thus, it is important to carefully characterize the royal jelly products used in intervention studies to ensure consistency and accuracy of results.

At now, the probable mechanism underlying the effect of royal jelly on anthropometric indices is unknown, and further investigation is necessary. Yet, some studies have hypothesized that a range of biological mechanisms may have a role in this regard. Royal jelly supplementation increases peroxisome proliferator-activated-alpha (PPAR-a) expression, enhancing lipolysis and contributing to a decrease in body weight (46). Moreover, royal jelly declines adiposity, induces beige phenotype in white adipose tissue, and activates brown adipose tissue thermogenic program via a significant up-regulation of UCP-1 as a marker protein of brown adipocytes concurrent with an increase in the expression of PRDM16; a primary modulator of BAT development; and P38MAPK, BMP8B, and CEREB1 as additional thermogenic components (47). Enhanced oxygen metabolism, respiration, and oxidative phosphorylation may be responsible for the favorable effects of royal jelly (25). Also, 10-HDA of royal jelly activates TRPA1 (transient receptor potential Ankyrin 1) and TRPV1 (vanilloid1) which in turn induces thermogenesis and increases energy expenditure (26). The hypocholesterolemic properties of royal jelly are primarily attributed to its proteins, particularly MRJP1 and MRJP2, which could potentially affect anthropometric indices. The peptide MRJP exhibits a notable capacity for binding bile acids and can effectively hinder cholesterol absorption by reducing the micellar solubility of cholesterol in the jejunum. Furthermore, the aforementioned characteristic of MRJP has the potential to impede the process of bile acid reabsorption in the ileum, ultimately leading to an escalation in the excretion of fecal steroids (48). Moreover, the supplementation of royal jelly in overweight adults increases leptin, a hormone with a significant role in maintaining a healthy weight by promoting feelings of fullness and regulating energy balance (42, 49).

### Strengths and limitations

The current study had notable strengths. To the best of our knowledge, our study was the first systematic review and meta-analysis investigated the effect of royal jelly on anthropometric indices. Only results from randomized, double-blinded clinical trials, which represent the highest level of clinical evidence, were included in this study. Second, we found no evidence of publication bias that could have affected the findings of a meta-analysis. Third, by conducting a comprehensive search of the literature and adhering to the PRISMA guidelines for conducting and reporting the review, we attempted to minimize any potential biases in the review process. Fourth, we assessed the overall quality of evidence using the GRADE system for each outcome. Fifth, subgroup analyses were conducted to examine the effects across subgroups and identify plausible sources of heterogeneity.

Regardless of the strengths mentioned, our study has some limitations needed to be considered. The main limitation of the study is the lack of any information about the quality of royal jelly products in studied populations, which can influence the final findings. Although we were able to pool the data from several studies in our meta-analysis, the overall sample size was still relatively small, which results in low statistical power. There are concerns about the heterogeneity of the included studies in terms of health status, dosage, and duration, which may have affected the efficacy of the results. Moreover, the majority of the included studies were conducted within a brief time frame (less than 6 months). So, we could not investigate the impact of royal jelly on anthropometric indices in long term. Finally, although physical activity and energy intake are among the major contributing factors in anthropometric indices, none of the included studies adjusted their result for changes in these parameters. Hence, we were unable to determine the net effect of royal jelly supplementation on anthropometric indices.

Finally, many of the included studies did not adjust their results for probable confounders; hence their effects were not taken into account.

## Conclusion

In conclusion, royal jelly supplementation had no significant influence on anthropometric indices including BW, BMI, and FM. However, based on subgroup analysis, our results revealed a significant reduction in BW and BMI following royal jelly intake in subgroup of royal jelly dosage <3,000 mg/day. Future RCTs with larger sample sizes, different BMI ranges, and longer duration of supplementation are warranted to confirm and enhance the precision of our findings.

## Data availability statement

The original contributions presented in the study are included in the article/Supplementary material, further inquiries can be directed to the corresponding authors.

## Ethics statement

The study protocol was approved and registered by the ethics committee of Isfahan University of Medical Sciences (identifier: IR.MUI.RESEARCH. REC.1402.014).

## Author contributions

MV, HB, and GA were the main researcher, designed the hypothesis, and supervised the project. The literature search and screening data were done by MD, VM, and MK. Data extraction and quality assessment were performed independently by MD, MV, NN, and MK. MV, NN, MD, GA, and ES analyzed and interpreted data and wrote the manuscript. All authors contributed to the article and approved the submitted version.

## Conflict of interest

The authors declare that the research was conducted in the absence of any commercial or financial relationships that could be construed as a potential conflict of interest.

## Publisher’s note

All claims expressed in this article are solely those of the authors and do not necessarily represent those of their affiliated organizations, or those of the publisher, the editors and the reviewers. Any product that may be evaluated in this article, or claim that may be made by its manufacturer, is not guaranteed or endorsed by the publisher.

## Supplementary material

The Supplementary material for this article can be found online at: https://www.frontiersin.org/articles/10.3389/fnut.2023.1196258/full#supplementary-material
