# Supplementary material for: The effects of royal jelly supplementation on anthropometric indices: a GRADE-assessed systematic review and dose-response meta-analysis of randomized controlled trials
Source: Front Nutr. 2023 Aug 3;10:1196258. doi: 10.3389/fnut.2023.1196258 (PMC10438987; doi:10.3389/fnut.2023.1196258)
Supplement: Supplementary file 1 [file Data_Sheet_1.DOCX]

Title: **The effects of Royal jelly supplementation on anthropometric indices: A GRADE-assessed systematic review and dose-response meta-analysis of randomized controlled trials**

### Mahdi Vajdi^1a^ and Vali Musazadeh^2a^, Mahsa Khajeh^1^, Ehsan Safaei^3^, Melika Darzi ^4^, Nooshin Noshadi^3^, Hadi Bazyar^5^**, Gholamreza Askari^6^*

^1^ Student Research Committee, Isfahan University of Medical Sciences, Isfahan, Iran

^2^ Student research Committee, Tabriz university of Medical sciences, Tabriz, Iran

3 Nutrition Research Center, Department of Clinical Nutrition, School of Nutrition and Food Sciences, Tabriz University of Medical Sciences, Tabriz, Iran

^4^ Department of Nutrition, Science and Research Branch, Islamic Azad University, Tehran, Iran

^5^ Department of Public Health, Sirjan School of Medical Sciences, Sirjan, Iran

^6^ Department of Community Nutrition, School of Nutrition and Food Science, Nutrition and Food Security Research Center, Isfahan University of Medical Science*,* Isfahan*, Iran*

**^a^Note:** Mahdi Vajdi and Vali Musazadeh have contributed equally to this work and share first authorship.

***Corresponding author:**

Gholamreza Askari

Department of Community Nutrition, School of Nutrition and Food Science, Nutrition and Food Security Research Center, Isfahan University of Medical Science*,* Isfahan*,* Iran, PO Box:81745, Isfahan, Iran

E-mail address: Askari@mui.ac.ir

** **Co-Corresponding author:**

Hadi Bazyar

Department of Public Health, Sirjan School of Medical Sciences, Sirjan, Iran

**Supplementary Material**

**Supplementary Table 1.** PRISMA Checklist

| **Section/topic** | **#** | | | **Checklist item** | **Reported on page #** |
| --- | --- | --- | --- | --- | --- |
| **TITLE** | | | | |  |
| Title | 1 | | | Identify the report as a systematic review, meta-analysis, or both. | Page 1 |
| **ABSTRACT** | | | | |  |
| Structured summary | 2 | | | Provide a structured summary including, as applicable: background; objectives; data sources; study eligibility criteria, participants, and interventions; study appraisal and synthesis methods; results; limitations; conclusions and implications of key findings; systematic review registration number. | Page 2 |
| **INTRODUCTION** | | | | |  |
| Rationale | 3 | | | Describe the rationale for the review in the context of what is already known. | Page 3 |
| Objectives | 4 | | | Provide an explicit statement of questions being addressed with reference to participants, interventions, comparisons, outcomes, and study design (PICOS). | Page 4 |
| **METHODS** | | | | |  |
| Protocol and registration | 5 | Indicate if a review protocol exists, if and where it can be accessed (e.g., Web address), and, if available, provide registration information including registration number. | | | Page 4 |
| Eligibility criteria | 6 | Specify study characteristics (e.g., PICOS, length of follow-up) and report characteristics (e.g., years considered, language, publication status) used as criteria for eligibility, giving rationale. | | | Page 5 |
| Information sources | 7 | Describe all information sources (e.g., databases with dates of coverage, contact with study authors to identify additional studies) in the search and date last searched. | | | Page 5 |
| Search | 8 | Present full electronic search strategy for at least one database, including any limits used, such that it could be repeated. | | | Page 5 |
| Study selection | 9 | State the process for selecting studies (i.e., screening, eligibility, included in systematic review, and, if applicable, included in the meta-analysis). | | | Page 5; |
| Data collection process | 10 | Describe method of data extraction from reports (e.g., piloted forms, independently, in duplicate) and any processes for obtaining and confirming data from investigators. | | | Page 5 |
| Data items | 11 | List and define all variables for which data were sought (e.g., PICOS, funding sources) and any assumptions and simplifications made. | | | Page 6 |
| Risk of bias in individual studies | 12 | Describe methods used for assessing risk of bias of individual studies (including specification of whether this was done at the study or outcome level), and how this information is to be used in any data synthesis. | | | Page 6 |
| Summary measures | 13 | State the principal summary measures (e.g., risk ratio, difference in means). | | | Page 6 |
| Synthesis of results | 14 | Describe the methods of handling data and combining results of studies, if done, including measures of consistency (e.g., I^2^) for each meta-analysis. | | | Page 6 |
| Risk of bias across studies | 15 | | Specify any assessment of risk of bias that may affect the cumulative evidence (e.g., publication bias, selective reporting within studies). | | Page 6 |
| Additional analyses | 16 | | Describe methods of additional analyses (e.g., sensitivity or subgroup analyses, meta-regression), if done, indicating which were pre-specified. | | Page 6 |
| **RESULTS** | | | | |  |
| Study selection | 17 | | Give numbers of studies screened, assessed for eligibility, and included in the review, with reasons for exclusions at each stage, ideally with a flow diagram. | | Page 7 |
| Study characteristics | 18 | | For each study, present characteristics for which data were extracted (e.g., study size, PICOS, follow-up period) and provide the citations. | | Page 7 |
| Risk of bias within studies | 19 | | Present data on risk of bias of each study and, if available, any outcome level assessment (see item 12). | | Fig2 |
| Results of individual studies | 20 | | For all outcomes considered (benefits or harms), present, for each study: (a) simple summary data for each intervention group (b) effect estimates and confidence intervals, ideally with a forest plot. | | Page 8 , 9 |
| Synthesis of results | 21 | | Present results of each meta-analysis done, including confidence intervals and measures of consistency. | | Figures 1-3 |
| Risk of bias across studies | 22 | | Present results of any assessment of risk of bias across studies (see Item 15). | | Figure 1 |
| Additional analysis | 23 | | Give results of additional analyses, if done (e.g., sensitivity or subgroup analyses, meta-regression [see Item 16]). | |  |
| **DISCUSSION** | | | | |  |
| Summary of evidence | 24 | | Summarize the main findings including the strength of evidence for each main outcome; consider their relevance to key groups (e.g., healthcare providers, users, and policy makers). | | Page 10 |
| Limitations | 25 | | Discuss limitations at study and outcome level (e.g., risk of bias), and at review-level (e.g., incomplete retrieval of identified research, reporting bias). | | Page 12 |
| Conclusions | 26 | | Provide a general interpretation of the results in the context of other evidence, and implications for future research. | | Page 12 |
| **FUNDING** | | | | |  |
| Funding | 27 | | Describe sources of funding for the systematic review and other support (e.g., supply of data); role of funders for the systematic review. | | Page 12 |

**Supplementary Table 2**. Results of subgroup analyses for the effects of royal jelly on body weight according to intervention or participant characteristics

| **Study group** | **Studies,**  ***n*** | **WMD (95%CI)^1^** | ***P*-effect** | ***P*-within group^2^** | ***I^2^* (%)^3^** | P for between subgroup heterogeneity | |
| --- | --- | --- | --- | --- | --- | --- | --- |
| **Sample size** |  |  |  |  |  | 0.483 |  |
| < 45 | 5 | -0.04 (-1.17, 1.08) | 0.27 | 0.631 | 0 |  |  |
| ≥ 45 | 3 | -0.51 (-1.19, 0.17) | 0.004 | 0.003 | 82.9 |  |  |
| **Dose** |  |  |  |  |  | 0.010 |  |
| <3000 | 4 | -1.23 (-2.10, -0.36) | 0.005 | 0.211 | 33.5 |  |  |
| ≥3000 | 4 | 0.31 (-0.47, 1.10) | 0.434 | 0.312 | 15.9 |  |  |
| **Duration** |  |  |  |  |  | 0.333 |  |
| <8 | 4 | 0.09 (-1.04, 1.24) | 0.865 | 0.990 | 0 |  |  |
| ≥8 | 4 | -0.55 (-1.23, 0.12) | 0.108 | 0.003 | 78.1 |  |  |
| **Sex** |  |  |  |  |  | 0.004 |  |
| Male | 3 | -0.01 (-1.44, 1.41) | 0.986 | 0.974 | 0 |  |  |
| Female | 1 | -1.95 (-3.04, -0.85) | <0.001 | - | - |  |  |
| Both | 4 | 0.31 (-0.47, 1.10) | 0.438 | 0.312 | 15.9 |  |  |
| **Health status** |  |  |  |  |  | 0.483 |  |
| Healthy | 5 | -0.04 (-1.17, 1.08) | 0.944 | 0.631 | 0 |  |  |
| T2DM | 3 | -0.51 (-1.19, 0.17) | 0.141 | 0.003 | 82.9 |  |  |
| **Quality** |  |  |  |  |  | 0.483 |  |
| Low | 5 | -0.04 (-1.17, 1.08) | 0.944 | 0.631 | 0 |  |  |
| High | 3 | -0.51 (-1.19, 0.17) | 0.141 | 0.003 | 82.9 |  |  |

^1^Obtained from the fixed-effects model, ^2^Refers to the mean (95% CI), ^3^Inconsistency, percentage of variation across studies due to heterogeneity. T2DM, type 2 diabetes

**Supplementary Table 3**. Results of subgroup analyses for the effects of royal jelly on BMI according to intervention or participant characteristics

| **Study group** | **Studies,**  ***n*** | **WMD (95%CI)^1^** | ***P*-effect** | ***P*-within group^2^** | ***I^2^* (%)^3^** | P for between subgroup heterogeneity | |
| --- | --- | --- | --- | --- | --- | --- | --- |
| **Sample size** |  |  |  |  |  | 0.377 |  |
| < 45 | 4 | -0.10 (-0.37, 0.16) | 0.466 | 0.768 | 0 |  |  |
| ≥ 45 | 6 | 0.02 (-0.05, 0.10) | 0.508 | <0.001 | 97.3 |  |  |
| **Dose** |  |  |  |  |  | <0.001 |  |
| <3000 | 6 | -0.33 (-0.45, -0.21) | <0.001 | <0.001 | 81.2 |  |  |
| ≥3000 | 4 | 0.22 (0.13, 0.32) | <0.001 | <0.001 | 97.2 |  |  |
| **Duration** |  |  |  |  |  | 0.465 |  |
| <8 | 5 | -0.04 (-0.21, 0.13) | 0.636 | 0.849 | 0 |  |  |
| ≥8 | 5 | 0.03 (-0.05, 0.11) | 0.481 | <0.001 | 97.8 |  |  |
| **Age** |  |  |  |  |  | 0.508 |  |
| <40 | 3 | -0.10 (-0.45, 0.25) | 0.579 | 0.587 | 0 |  |  |
| ≥40 | 7 | 0.02 (-0.05, 0.09) | 0.573 | <0.001 | 96.7 |  |  |
| **Sex** |  |  |  |  |  | <0.001 |  |
| Male | 3 | -0.10 (-0.45, 0.25) | 0.579 | 0.587 | 0 |  |  |
| Female | 1 | -0.68 (-0.86, -0.49) | <0.001 | - | - |  |  |
| Both | 6 | 0.174 (0.08, 0.25) | <0.001 | <0.001 | 95.6 |  |  |
| **Health status** |  |  |  |  |  | 0.193 |  |
| Healthy | 6 | 0.06 (-0.02, 0.15) | 0.175 | 0.664 | 0 |  |  |
| T2DM | 3 | -0.08 (-0.23, 0.06) | 0.258 | <0.001 | 98.9 |  |  |
| Overweight | 1 | -0.10 (-0.42, 0.22) | 0.550 | - | - |  |  |
| **Quality** |  |  |  |  |  | 0.377 |  |
| Low | 4 | -0.10 (-0.37, 0.16) | 0.466 | 0.786 | 0 |  |  |
| High | 6 | 0.02 (-0.05, 0.10) | 0.508 | <0.001 | 97.3 |  |  |

^1^Obtained from the fixed -effects model, ^2^Refers to the mean (95% CI), ^3^Inconsistency, percentage of variation across studies due to heterogeneity. T2DM, type 2 diabetes

**Supplementary Table 4**. Results of subgroup analyses for the effects of royal jelly on fat mass percent according to intervention or participant characteristics

| **Study group** | **Studies,**  ***n*** | **WMD (95%CI)^1^** | ***P*-effect** | ***P*-within group^2^** | ***I^2^* (%)^3^** | P for between subgroup heterogeneity | |
| --- | --- | --- | --- | --- | --- | --- | --- |
| **Sample size** |  |  |  |  |  | 0.311 |  |
| < 45 | 4 | 0.18 (-0.35, 0.71) | 0.502 | 0.500 | 0 |  |  |
| ≥ 45 | 1 | -0.30 (-1.06, 0.46) | 0.443 | - | - |  |  |
| **Dose** |  |  |  |  |  | 0.463 |  |
| <3000 | 4 | 0.08 (-0.38, 0.55) | 0.713 | 0.415 | 0 |  |  |
| ≥3000 | 1 | -0.40 (-1.61, 0.81) | 0.519 | - | - |  |  |
| **Duration** |  |  |  |  |  | 0.311 |  |
| <8 | 4 | 0.18 (-0.35, 0.71) | 0.502 | 0.500 | 0 |  |  |
| ≥8 | 1 | -0.30 (-1.06, 0.46) | 0.443 | - | - |  |  |
| **Age** |  |  |  |  |  | 0.147 |  |
| <40 | 3 | 0.32 (-0.27, 0.91) | 0.288 | 0.530 | 0 |  |  |
| ≥40 | 2 | -0.32 (-0.97, 0.32) | 0.321 | 0.892 | 0 |  |  |
| **Sex** |  |  |  |  |  | 0.147 |  |
| Male | 3 | 0.32 (-0.27, 0.91) | 0.288 | 0.530 | 0 |  |  |
| Both | 2 | -0.32 (-0.97, 0.32) | 0.321 | 0.892 | 0 |  |  |
| **Health status** |  |  |  |  |  | 0.311 |  |
| Healthy | 4 | 0.18 (-0.35, 0.71) | 0.502 | 0.500 | 0 |  |  |
| Overweight | 1 | -0.30 (-1.06, 0.46) | 0.443 | - | - |  |  |
| **Quality** |  |  |  |  |  | 0.311 |  |
| Low | 4 | 0.18 (-0.35, 0.71) | 0.502 | 0.500 | 0 |  |  |
| High | 6 | -0.30 (-1.06, 0.46) | 0.443 | - | - |  |  |

^1^Obtained from the fixed-effects model, ^2^Refers to the mean (95% CI), ^3^Inconsistency, percentage of variation across studies due to heterogeneity. T2DM, type 2 diabetes


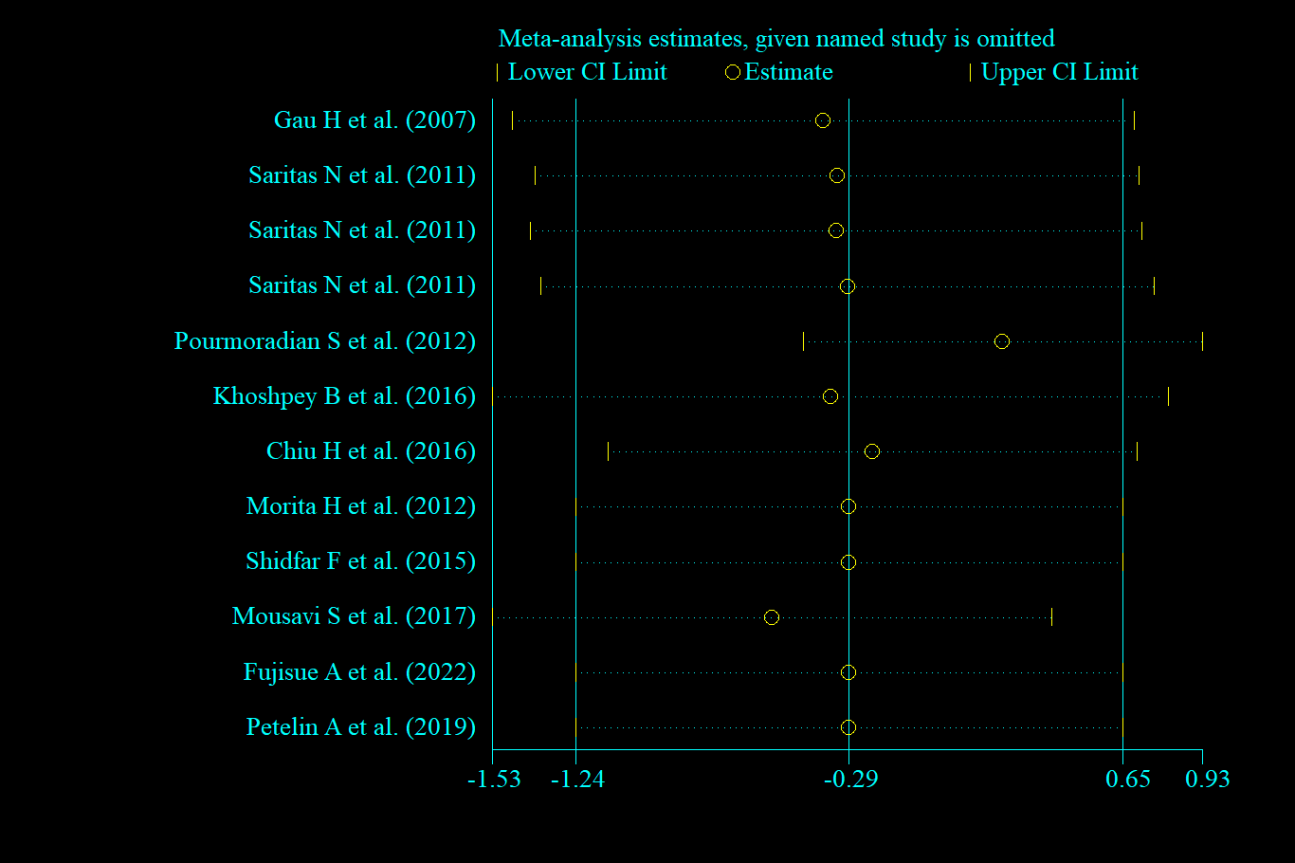


**Supplementary Figure 1.** Sensitivity analysis of royal jelly on body weight

A


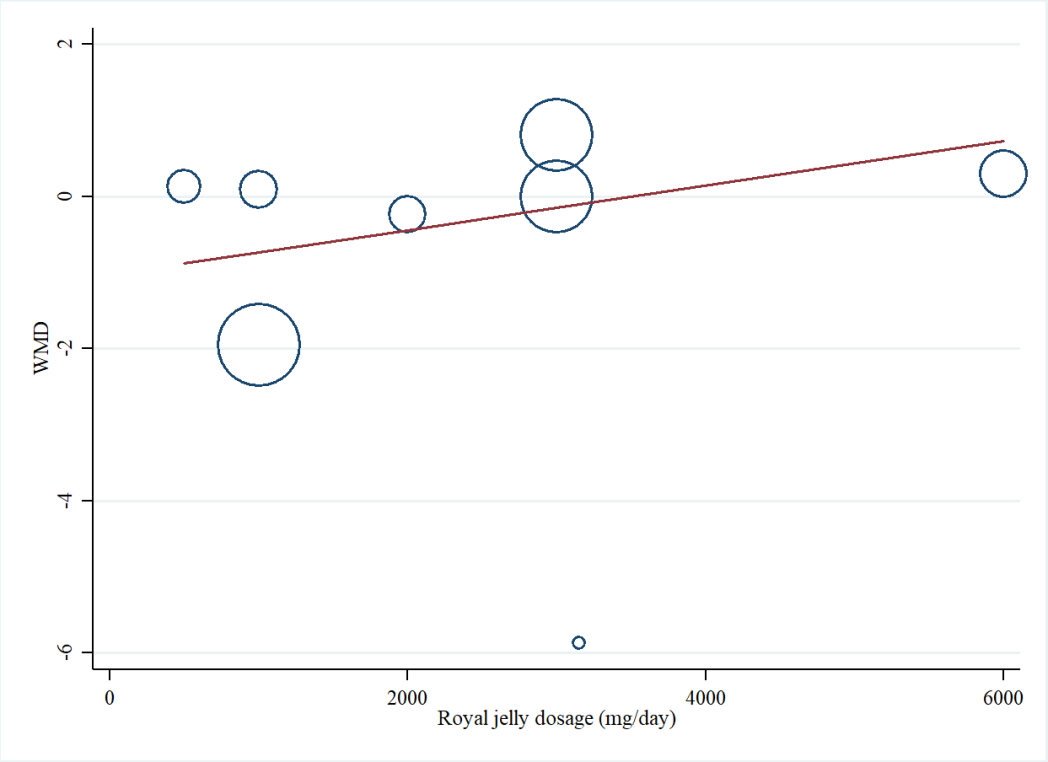


B


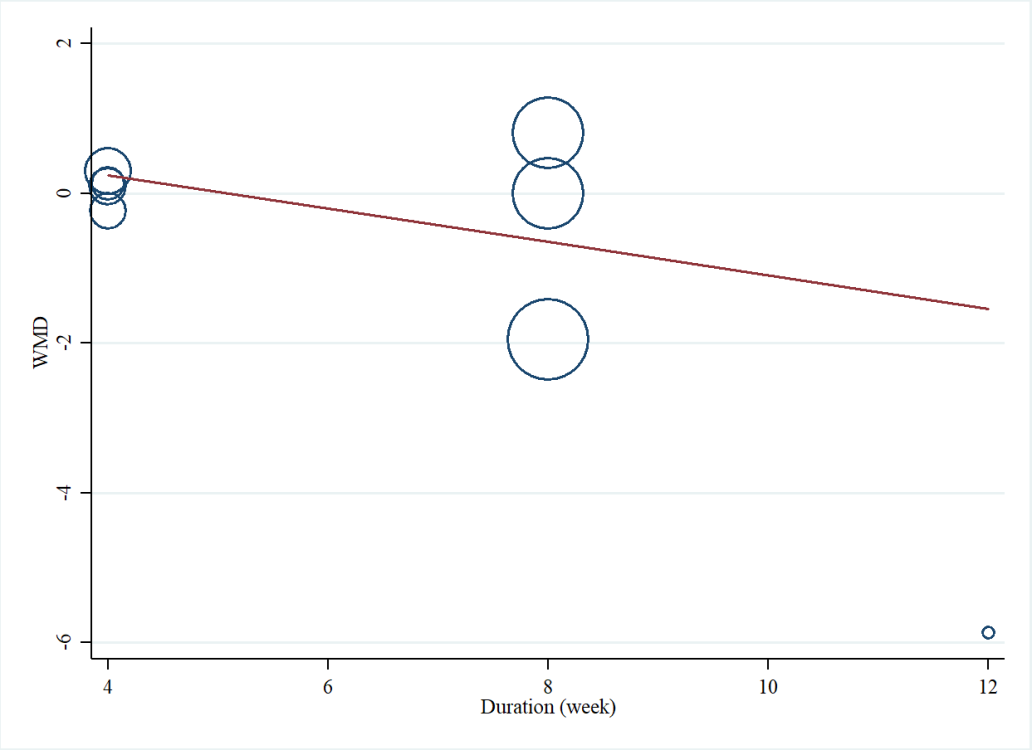


**Supplementary Figure 2.** Random-effects meta-regression plots of the association between mean changes in body weight and royal jelly dose (A) and intervention duratyion (B)


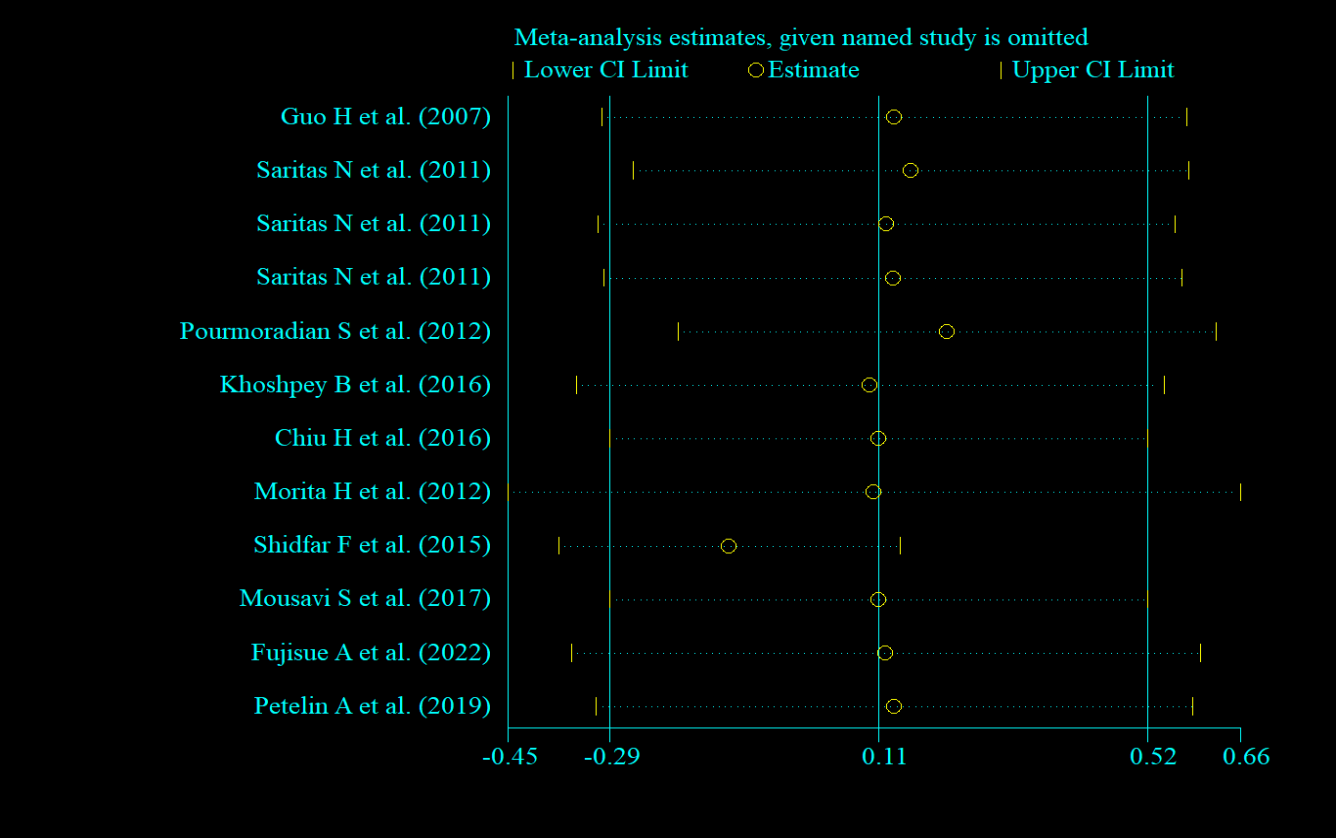


**Supplementary Figure 3.** Sensitivity analysis of royal jelly on BMI

A


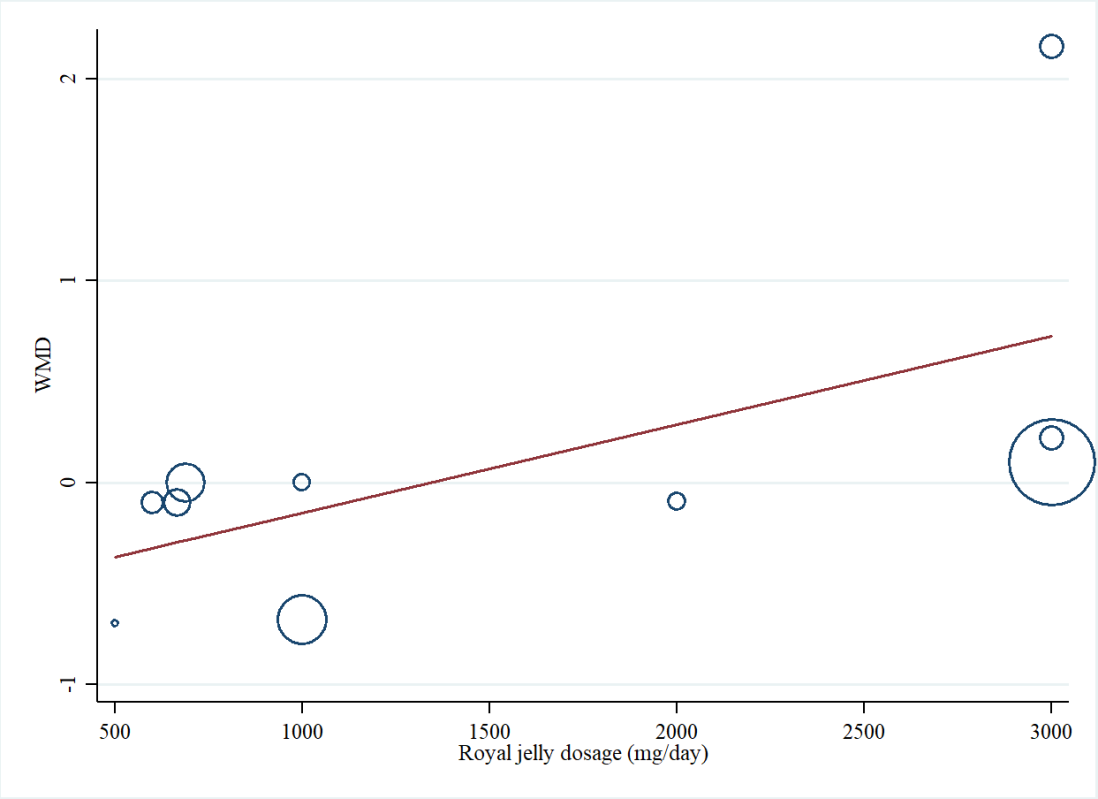


B


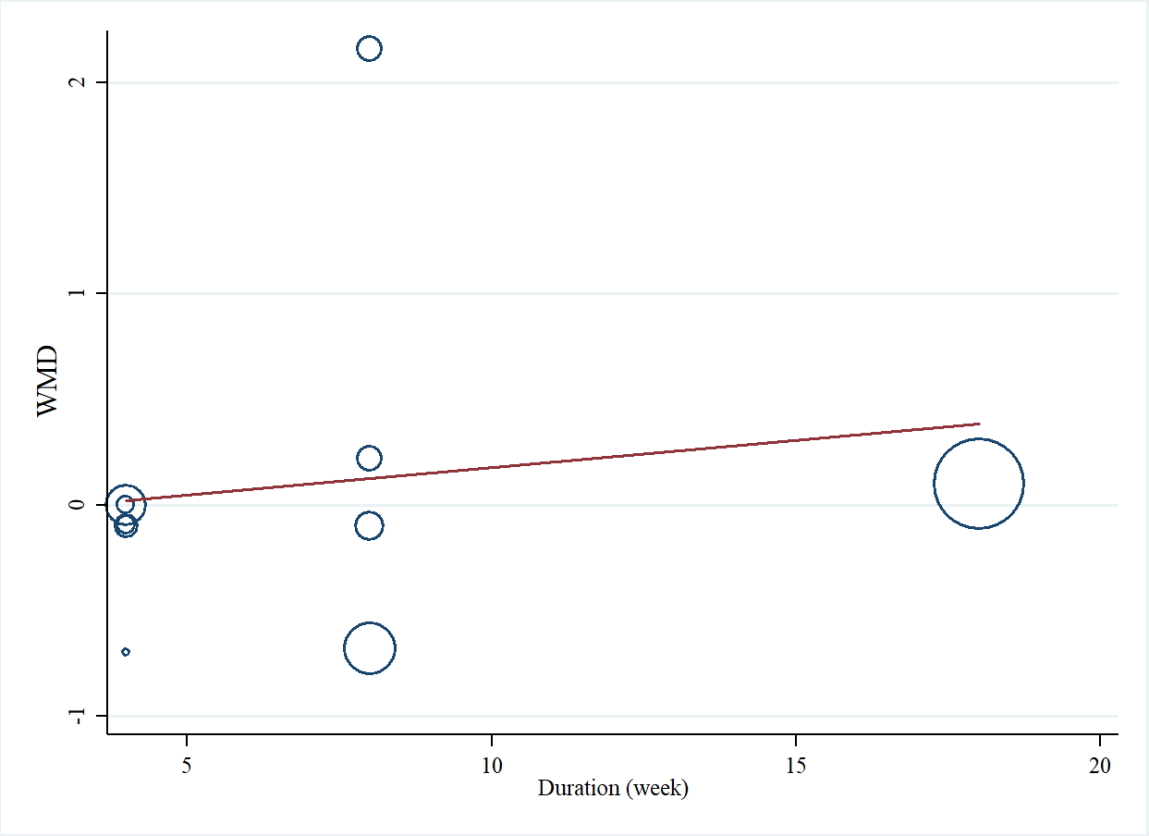


**Supplementary Figure 4.** Random-effects meta-regression plots of the association between mean changes in BMI and royal jelly dose (A) and intervention duratyion (B)

**
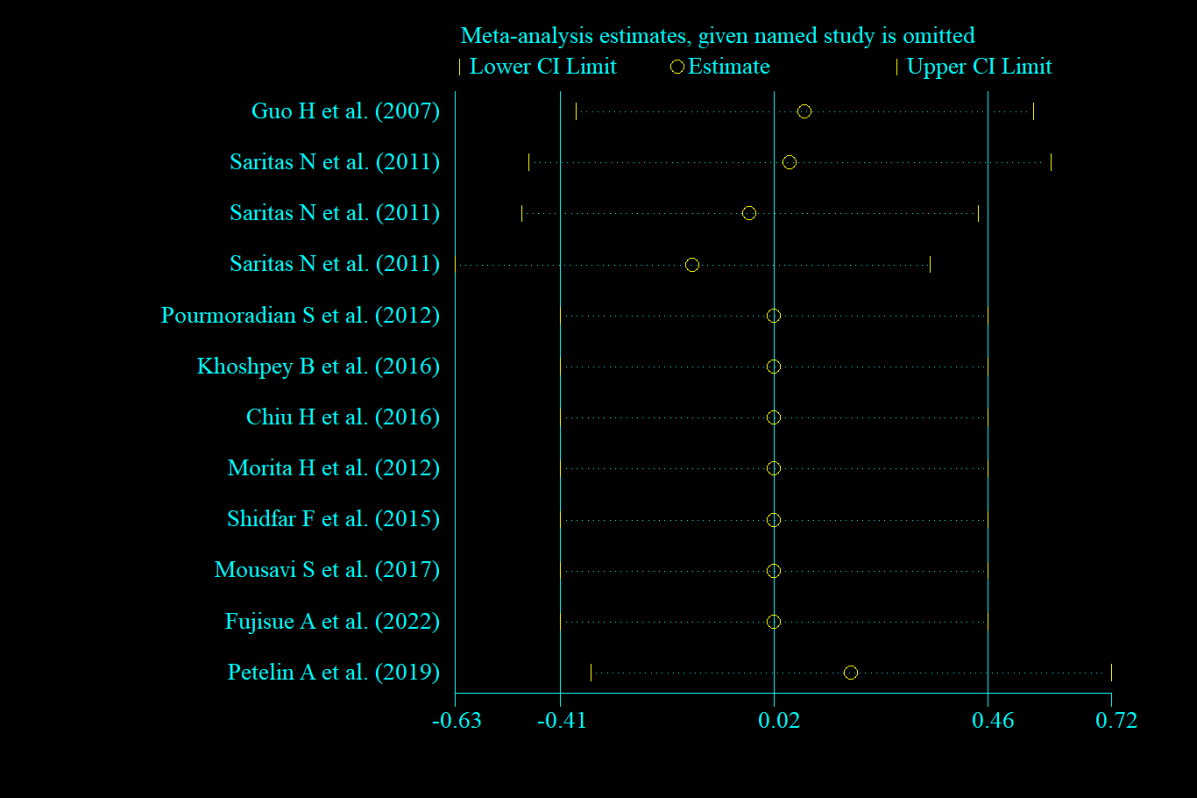
**

**Supplementary Figure 5.** Sensitivity analysis of royal jelly on fat mass

A


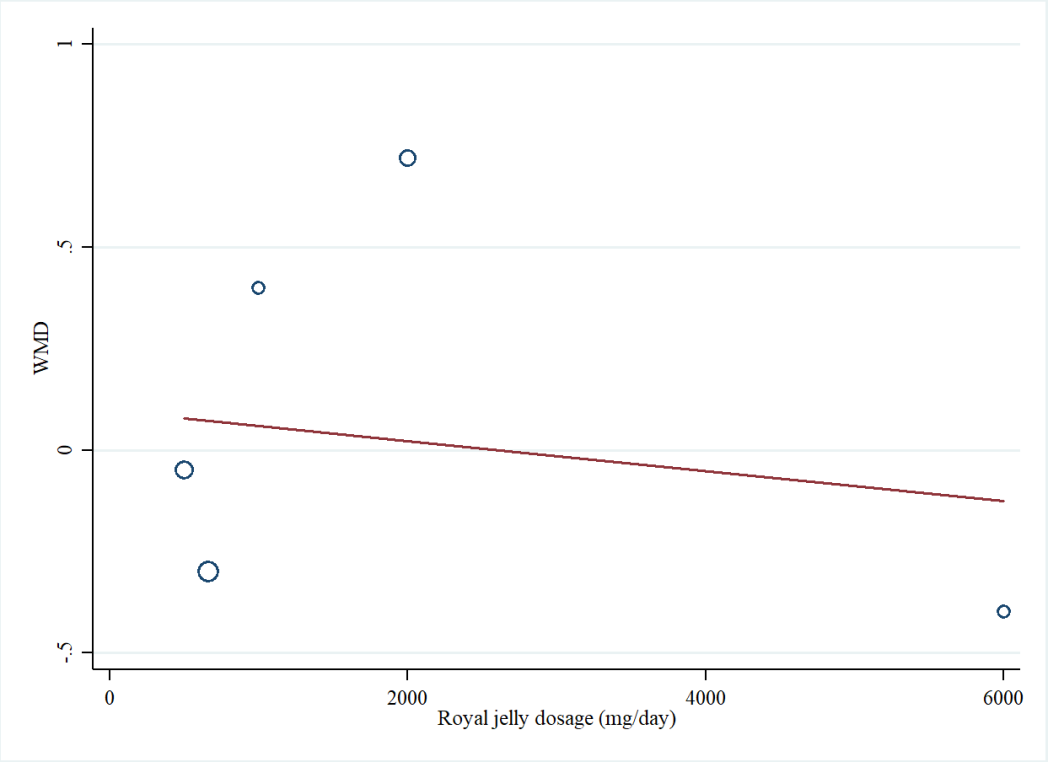


B


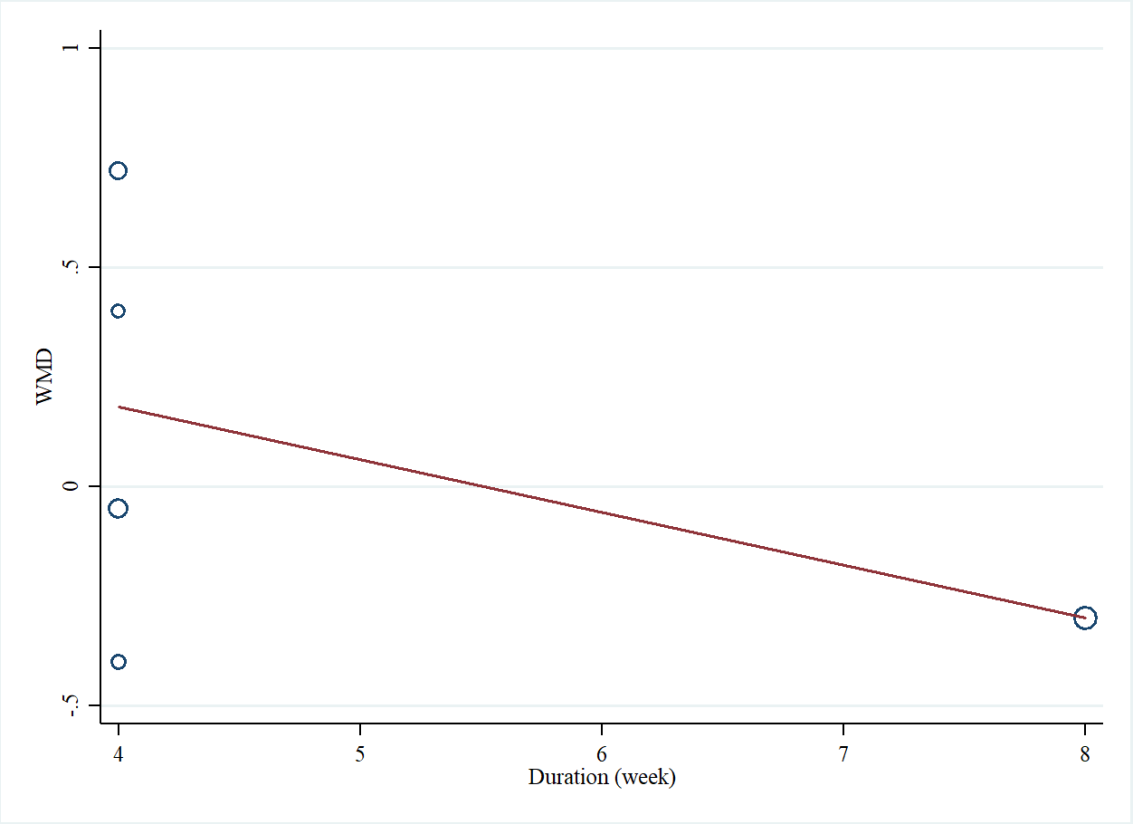


**Supplementary Figure 6.** Random-effects meta-regression plots of the association between mean changes in fat mass and royal jelly dose (A) and intervention duratyion (B)

**(a)**

**
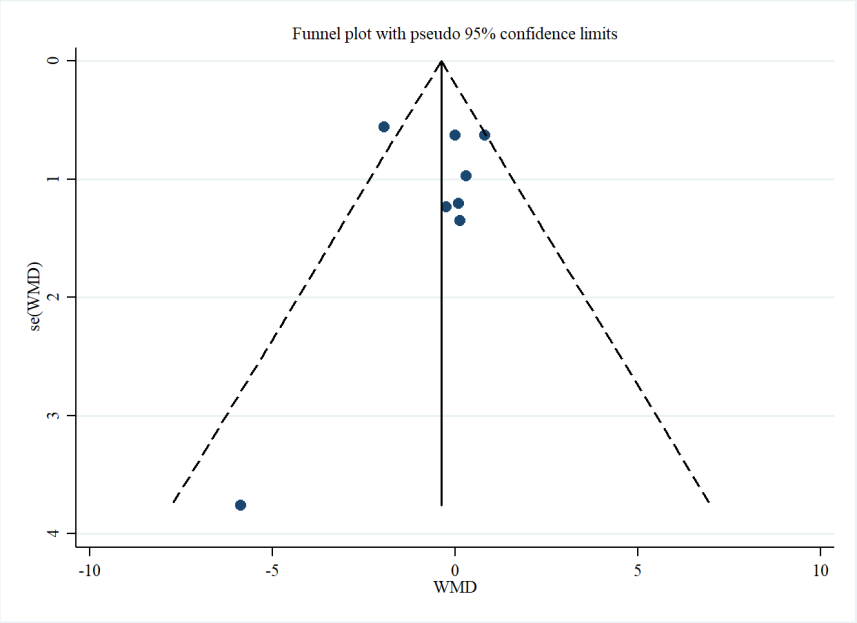
**

**(b)**


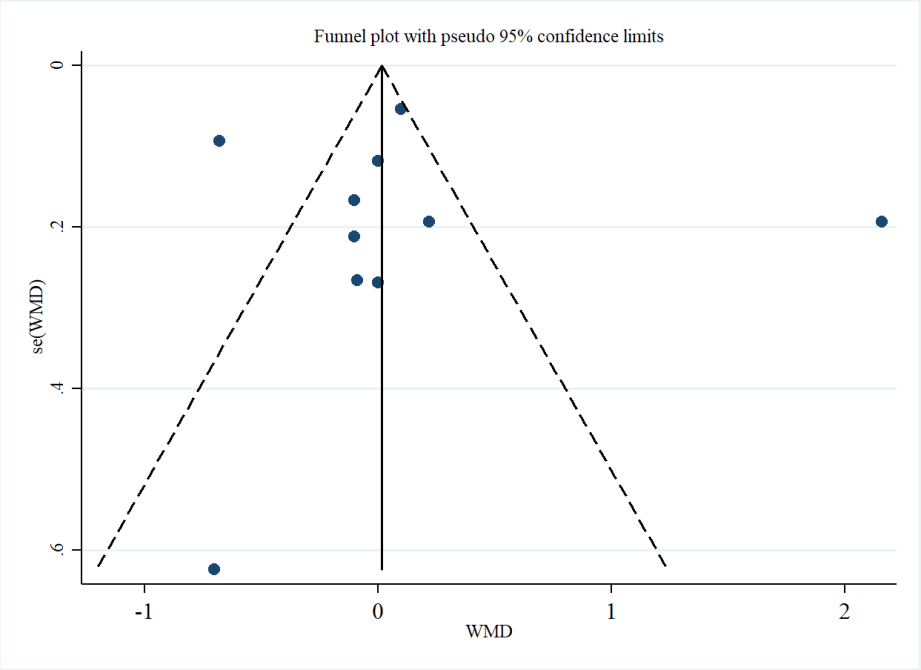


(c)


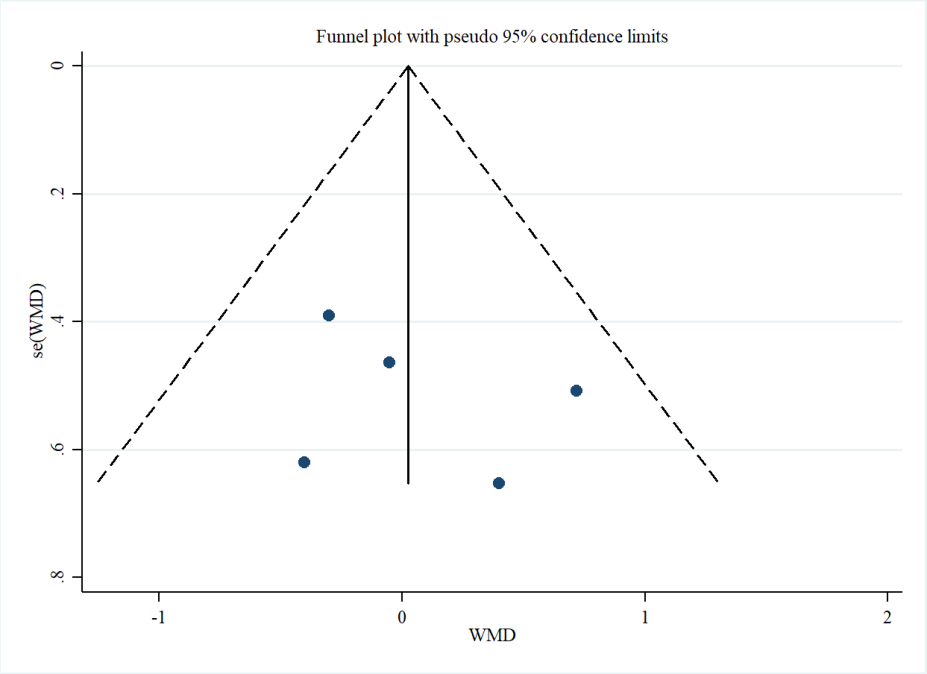


**Supplementary Figure 7.** Funnel plot for evaluation publication bias in the studies reporting the effect of royal jelly supplementation on BW (a), BMI (b), and fat mass (c)
